# Variation in genes involved in epigenetic processes offers insights into tropically adapted cattle diversity

**DOI:** 10.3389/fgene.2014.00089

**Published:** 2014-04-22

**Authors:** Laercio R. Porto-Neto, Marina R. S. Fortes, Sean M. McWilliam, Sigrid A. Lehnert, Antonio Reverter

**Affiliations:** ^1^CSIRO Food Futures Flagship and CSIRO Animal, Health and Food SciencesBrisbane, QLD, Australia; ^2^QAAFI, Centre for Animal Science, The University of QueenslandBrisbane, QLD, Australia

**Keywords:** epigenetics, polymorphisms, bovine species, cattle diversity, high-density genotypes

## Abstract

We evaluated the relevance of the BovineHD Illumina SNP chip with respect to genes involved in epigenetic processes. Genotypes for 729,068 SNP on two tropical cattle breeds of Australia were used: Brahman (*n* = 2112) and Tropical Composite (*n* = 2550). We used data mining approaches to compile a list of bovine protein-coding genes involved in epigenetic processes. These genes represent 9 functional categories that contain between one (histone demethylases) and 99 (chromatin remodeling factors) genes. A total of 3091 SNP mapped to positions within 3000 bp of the 193 coding regions of those genes, including 113 SNP in transcribed regions, 2738 in intronic regions and 240 in up- or down-stream regions. For all these SNP categories, we observed differences in the allelic frequencies between Brahman and Tropical Composite cattle. These differences were larger than those observed for the entire set of 729,068 SNP (*P* = 1.79 x 10^−5^). A multidimensional scaling analysis using only the 113 SNP in transcribed regions allowed for the separation of the two populations and this separation was comparable to the one obtained with a random set of 113 SNP (Principal Component 1 *r*^2^ > 0.84). To further characterize the differences between the breeds we defined a gene-differentiation metric based on the average genotypic frequencies of SNP connected to each gene and compared both cattle populations. The 10% most differentiated genes were distributed across 10 chromosomes, with significant (*P* < 0.05) enrichment on BTA 3 and 10. The 10% most conserved genes were located in 12 chromosomes. We conclude that there is variation between cattle populations in genes connected to epigenetic processes, and this variation can be used to differentiate cattle breeds. More research is needed to fully characterize the use of these SNP and its potential as means to further our understanding of biological variation and epigenetic processes.

## Introduction

Differences in gene transcriptional regulation between individuals are likely to contribute to quantitative variation in important livestock production traits such as growth, metabolic efficiency, immunological competence, and reproductive performance. A recent example is the mutation on the *Bos taurus* autosome (BTA) 14 that regulates the expression of various genes in the *PLAG1* region and is associated with many production traits as well as reproductive phenotypes (Karim et al., 2011; Fortes et al., 2013). Epigenetic modifications of DNA and of histone proteins contribute to the regulation of tissue- and developmental stage-specific gene expression.

Variations in transcriptional activity have been linked to a number of epigenetic processes including: differences in DNA methylation (Doerfler, 1983), the incorporation of histone variants into chromatin (Jin et al., 2009), histone modifications such as methylation and acetylation (Barth and Imhof, 2010), and Polycomb Group (PcG) proteins (Leeb et al., 2010). The epigenetic machinery that underpins these dynamic changes includes enzymes that catalyze addition or removal of methyl or acetyl groups to DNA or histone proteins [DNA methyltransferases (DNMT), histone methyltransferases (HMT) and histone acetyltransferases (HAT)], and chromatin remodeling factors.

In an example with promising relevance to livestock productivity, Lomniczi et al. (2013) have shown that the timing of female puberty in rats is driven by transcriptional activation of the *KISS1* promoter in the hypothalamus in a complex interplay of several components of the epigenetic machinery: DNA methylation, PcG proteins, and histone modifications. It is plausible that fertility traits in livestock may be subject to the same or similar regulatory mechanisms. Individual or breed differences in the epigenetic machinery could bring about changes that could, for example manifest themselves in an earlier age at puberty. Regulatory transcription factors associated with cattle puberty and heifer pregnancy have been proposed previously (Fortes et al., 2010, 2011, 2012).

DNA variations can affect regulatory elements of gene expression (Dunham et al., 2012). Adding complexity to this problem, recently DNA variations have been implicated in differential pattern of gene expression via epigenetic modulation (Furey and Sethupathy, 2013). Allele-specific activity of histone post-translational modifications affects transcriptional and chromatin activities (Kilpinen et al., 2013). Polymorphisms in transcription factor binding sites are, in some cases, causally responsible for alteration in histone marking (Kasowski et al., 2013; McVicker et al., 2013). Importantly, these alterations are largely transmitted from parent to offspring.

Cancer researchers have provided a number of recent examples where altered epigenetic patterns can be linked back to genetic alterations in the epigenetic machinery itself (Miremadi et al., 2007; Weichenhan and Plass, 2013). While these examples describe mutations that result in disease, it is plausible that subtle changes in the epigenetic machinery that affect quantities, binding affinities or target specificities of one of the elements could lead to slight shifts in gene regulations that can manifest as quantitative trait differences.

We used two populations of *Bos indicus* influenced cattle to investigate the genetic variability within epigenetic genes. We focused on this type of beef cattle because they are the most frequent in tropical and sub-tropical regions, corresponding to the largest proportion of beef production in the world (Herrero et al., 2013). *Bos indicus* cattle are much more tolerant to the tropical environment than are *Bos taurus* cattle, and *Bos indicus* × *Bos taurus* crosses often present at intermediate tolerance levels. However, once cattle crosses are bred under tropical conditions, natural and human-oriented selection starts to shape the herd speciation. Using these contrasting populations we aimed to explore if (1) there are SNP variations linked to genes belonging to epigenetic processes in a commercially available genotyping platform, if (2) the allelic frequency of these SNP varies between tropically adapted cattle breeds, and if (3) we could use these SNP to characterize the genetic diversity of cattle populations.

## Materials and methods

### Cattle and genotypes

The cattle were sourced from two populations, a *Bos indicus* (Brahman, *n* = 2112) and a cross-bred *Bos taurus* × *Bos indicus* (Tropical Composite, *n* = 2550) (Barwick et al., 2009). These animals were genotyped using either the BovineHD (Illumina, San Diego, California; http://res.illumina.com/documents/products/datasheets/datasheet_bovinehd.pdf) or the BovineSNP50 (Illumina, San Diego, California) (Matukumalli et al., 2009). Genotypes acquired with the lower density panel were imputed to higher density using Beagle (Browning and Browning, 2011). The imputation process and quality control applied to genotypes were described previously (Bolormaa et al., 2013). In brief, imputations were done within breed applying 30 iterations of Beagle using related individuals that were genotyped using the BovineHD as reference. SNP and individuals were filtered using stringent quality control parameters. All SNP were mapped to the UMD 3.1 assembly of the bovine genome sequence up-dated from Zimin et al. (2009) (http://www.cbcb.umd.edu/research/bos_taurus_assembly.shtml).

### Genes and transcripts involved in epigenetic processes

Data mining based on literature information and publicly available databases served to compile a list of 217 bovine protein-coding genes (and their transcript variants) involved in 9 categories of epigenetic processes (Esteller, 2006; Miremadi et al., 2007; Lomniczi et al., 2013; http://www.bioguo.org/AnimalTFDB/index.php). Similar data mining could be done for other species. The gene list used in this study is not intended to be comprehensive, but representative of the main biological processes with epigenetic consequences (Table 1). Where a gene had more than one transcript described, Ensemble tags (http://www.ensembl.org) were used as unique identifiers. The resulting list of genes and transcripts was the target for SNP selection.

**Table 1:** **Epigenetic categories, number of transcripts tagged, and number of SNP tagging these transcripts**.

**Epigenetic category**	**Gene function**	**Number of transcripts tagged**	Number of SNP tagging transcripts^*^		
			**CAT1**	**CAT2**	**CAT3**
a	DNA methyltransferases (DNMT)	3	2	33	6
b	Methyl-binding domain proteins (MBD)	6	2	43	8
c	Histone acetyltransferases (HAT)	11	8	164	13
d	Histone deacetylases (HDAC)	8	2	318	9
e	Histone methyltransferases (HMT)	32	26	637	37
f	Histone demethylase	1	0	12	2
g	Histones	30	3	18	43
h	Polycomb-group proteins	3	0	14	6
i	Chromatin-remodeling factors	99	70	1499	116
	TOTAL	193	113	2738	240

* CAT1, SNP at transcribed regions (exon, splice region, 3'- and 5'-UTR); CAT2, SNP at transcripts intron; CAT3, SNP down- or up-stream of a transcript (up to 3 Kb).

### SNP selection and annotation

We used four sources of information for SNP selection and annotation: (1) the annotation file of coding and non-coding transcripts (ftp://ftp.ensembl.org/pub/release-73/gtf/bos_taurus), (2) the list of targeted genes and transcripts, (3) the BovineHD SNP list, and (4) the Variant Effect Predictor (http://asia.ensembl.org/info/docs/tools/vep/index.html, *Bos taurus* release 73), all mapped using the bovine UMD 3.1 assembly as reference sequence. First we identified all SNP from the BovineHD (Illumina, San Diego) located at targeted transcripts or within 3Kb up- or downstream. Then, using the Variant Effect Predictor, we defined each SNP position relative to its target transcript and classify them in three categories accordingly: CAT1—corresponding to SNP mapped to exons, splice site, 3′ and 5′ UTR (transcribed SNP); CAT2—corresponding to intronic SNP; and CAT3—for SNP located up- or down-stream to selected transcripts.

### Population and gene diversity

Allelic and genotypic frequencies for all selected SNP (reference allele A) were calculated for each breed using PLINK v1.07 (Purcell et al., 2007). To further characterize the genetic differences between breeds using the target genes we applied a gene-differentiation metric derived from the Euclidean distance according to the following formulae:

$$\begin{array}{l} gene-differentiation={\left(\frac{{\text{p0}}_{\text{BB}}}{100}-\frac{{\text{p0}}_{\text{TC}}}{100}\right)}^{2}+{\left(\frac{{\text{p1}}_{\text{BB}}}{100}-\frac{{\text{p1}}_{\text{TC}}}{100}\right)}^{2}\\ \;+{\left(\frac{{\text{p2}}_{\text{BB}}}{100}-\frac{{\text{p2}}_{\text{TC}}}{100}\right)}^{2} \end{array}$$

Where p0, p1, and p2 are the average genotypic frequencies for all SNP within each gene (homozygous A, heterozygous and homozygous B) within each breed, and BB and TC are the Brahman and Tropical Composite genotypic frequencies, respectively. The hypergeometric test was applied to evaluate the significance of the distributions of individual allelic frequency categories and also to gene-differentiation metric. The hypergeometric test was set as the number of occurrences in one domain vs. the number of occurrences in another domain, e.g., number of SNP with allelic frequency difference between BB and TC > 0.3 within all SNP vs. the same situation within epigenetic SNP. The population differentiation was also evaluated using multidimensional scaling analyses, implemented in PLINK, using only the SNP at transcribed regions (CAT1, *n* = 113), and comparing its result to the average value obtain after 10 runs of 113 transcribed SNP randomly selected from the entire HD dataset.

## Results and discussion

**Figure 1:**
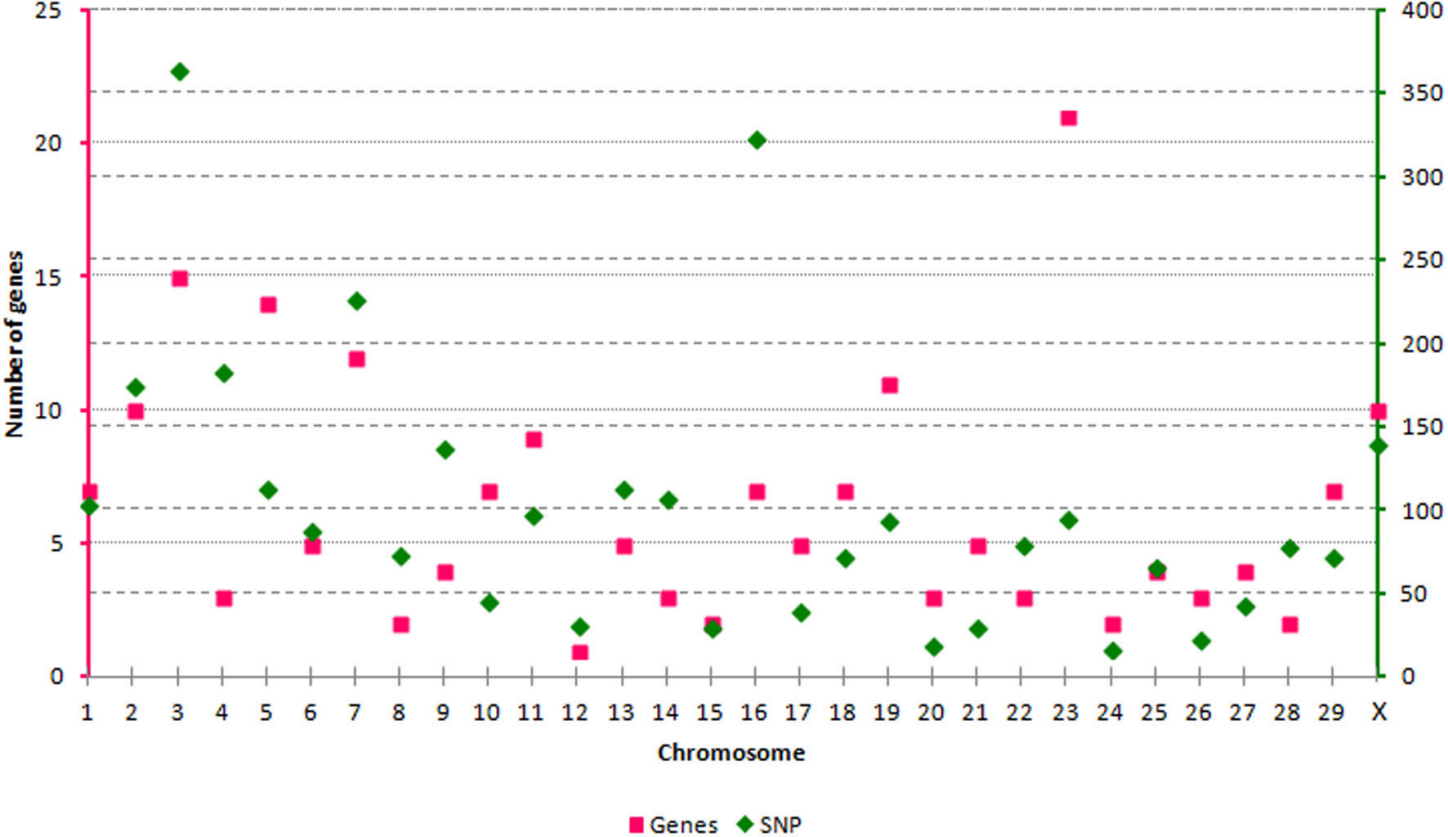
**Distribution of target genes and SNP along the genome**.

We have evaluated the possibility of using SNP from the BovineHD panel to tag genes and transcripts involved in epigenetic processes. Out of the 729,068 SNP available, 3091 SNP could be linked to an epigenetic gene. All autosomes and the X chromosome were represented by a varying number of SNP per chromosome, from *n* = 17 (BTA24) to *n* = 364 (BTA3) (Figure 1). The SNP were split in 3 categories according to their position in relation to the transcript tagged. The SNP in CAT1 were located in an exon, or the splice region, or 3′- and 5′-UTR (*n* = 113). The SNP in CAT2 were intronic (*n* = 2738) and in CAT3 were down- or up-stream of a transcript (up to 3 Kb) (*n* = 240). In order to define unique pairs of transcripts and SNP, only the closest transcript was mapped against each SNP. Therefore, if two transcripts mapped close together, and one of them had an intronic SNP and the next SNP was more than 3 Kb distant from the coding sequence, only the transcript with an intronic SNP was tagged. This step might have reduced the number of genes and transcripts that were tagged, but was needed to define unique SNP-gene pairs. As a result, 193 members of the target epigenetic transcripts contained SNP from BovineHD at or close by their coding sequence (Supplementary Table 1). These were classified into nine categories related to the biological processes in which they are involved (Table 1). The chromatin-remodeling factor category was the largest, with 99 transcripts, while the histone demethylase category was represented by just one transcript (*n* = 1). All main biological processes with potential direct epigenetic consequence were represented by at least one gene and 14 SNP. Hence, the BovineHD panel can be used to retrieve information regarding genes related to epigenetic processes.

Allelic frequency differences between Brahman and Tropical Composites were observed for all gene- and SNP-categories across the genome. The number of SNP with allelic frequency differences > 0.3 within the selected SNP were larger than those observed for the entire dataset (*P* = 1.79 × 10^−5^). Moreover, breaking the analyses by epigenetic SNP-categories, all had significant (*P* < 0.05) accumulation of SNP with allelic frequency difference > 0.3 between breeds, with higher significance for the CAT1 (transcribed SNP, *P* = 0.005) and CAT2 (intronic, *P* = 0.001). For gene-SNP categories that had more than 10 SNP frequencies were also calculated. These averages carried large standard deviation. Nevertheless, the top 3 averages of allelic frequency differences were for intronic SNP (CAT2) at methyl-binding domain proteins, polycomb-group proteins and transcribed SNP (CAT1) at Chromatin-remodeling factors. In contrast, the top 3 averages for allelic similarity were for intronic SNP (CAT2) at histones, histone demethylases, histone deacetylases. Overall, there was a significant accumulation of SNP with divergent allelic frequency between the studied cattle breeds, and often these SNP were located in potentially functional sites within the genes involved in epigenetic processes. It has been described that several epigenetic regulatory elements are evolutionary conserved across species (Xiao et al., 2012; Long et al., 2013), nevertheless we observed large variability between these two populations, which are very closely related, in evolutionary terms. The markers selected here *per se* may not necessarily be functionally active. However, their genome location and variability defines potential power for identification of variability on regulatory elements linked to functional DNA mutation.

**Figure 2:**
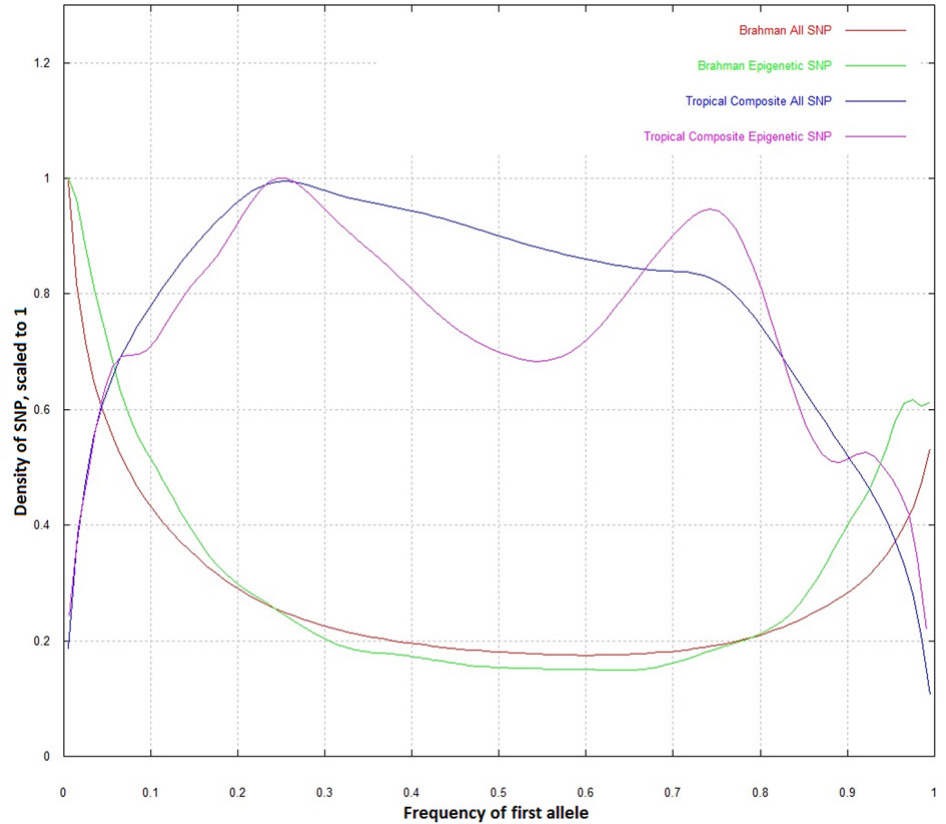
**Density plot of allelic frequencies for all SNP and epigenetic SNP in Brahman and Tropical Composite**.

When the whole dataset was compared to the epigenetic SNP, the distribution of allele frequencies in Brahman did not differ (Figure 2). However, in the Tropical Composite, there was an observable difference in which the epigenetic SNP dataset had a lower density at intermediate allelic frequencies, tending to be closer to the Brahman frequencies than non-epigenetic SNP. It seems that some “Brahman alleles” of some epigenetic SNP have been potentially selected within Tropical Composite. The Brahman cattle is mostly of *Bos indicus* origin while the Tropical Composite presents variable proportions of *Bos indicus* and *Bos taurus* ancestry (Porto-Neto et al., 2013). Considering that both types of *Bos* have many highly divergent phenotypes including climate adaptation, one could speculate that some of the markers with allelic frequency differences could be associated to these diverged phenotypes, and going a step further, could postulate that DNA variants linked to epigenetic process are potentially associated to environmental adaptation. Further research, including SNP association testing, is needed to support such speculations.

Multidimensional scaling calculated using only transcribed SNP (CAT1, *n* = 113) defined clear separation between breeds. The breeds clustering resulting from this analysis was comparable to that of other sets of randomly selected SNP (*n* = 113). The correlation of Component 1 between CAT1 and the average of 10 runs of randomly selected SNP being *r*^2^ = 0.91. There is enough variability between both cattle breeds to define independent clusters in a multidimensional analyses using 113 SNP and this variation is also extended to epigenetic-related SNP.

**Figure 3:**
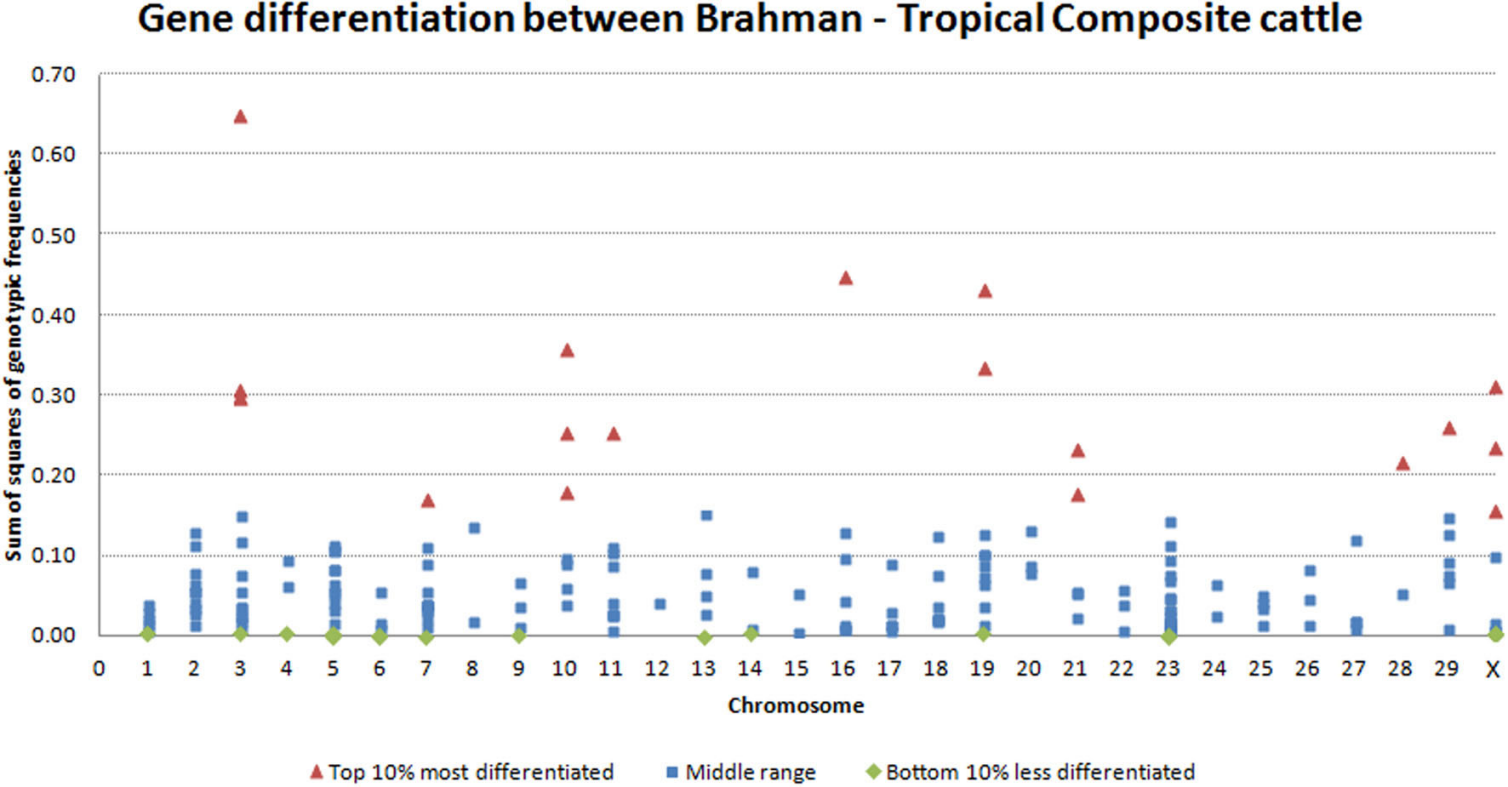
**Distribution of genes along the genome ranked by gene-differentiation metric**.

The variability observed in allelic frequency was also echoed in the gene-differentiation analysis. Within transcript, the comparison of averaged genotypic frequencies in each breed clearly showed differences. The top 10% most differentiated genes were distributed across 10 chromosomes, with significant (*P* = 0.05) concentration on BTA3 and BTA10. On the other hand, the top 10% most conserved genes were distributed in 12 chromosomes (Figure 3). Interestingly, some of the different splicing variants or different transcripts of the same gene had highly diverged gene-differentiation metric. For example, histone 4 has some transcripts at lower, some at middle and some at the top of the distribution for gene-differentiation estimates. Genetic elements of epigenetic processes are known to have redundancies and specificities in biological pathways (Comai, 2005; Haberland et al., 2009). Here, the observed divergence between transcripts of the same gene could characterize such redundancy that is an adaptive mechanism to temper the impact of specific gene variations on vital epigenetic processes. On the other hand, this divergence could demonstrate the plasticity of the genome in face of a diverse environment.

DNA variants can alter epigenetic processes, and also assist genomic mapping of epigenetic variance. Recently, the definition of epigenetics as changes in gene activity without changes in the DNA has been opened for re-consideration (Furey and Sethupathy, 2013). For example, it has been shown, based on histone modifications, that DNA variants in regulatory regions are often linked to active/repressed stated of genes (Kasowski et al., 2013), and that variations in transcription factors binding sites can mediate gene expression via its effect on histone modifications (Kilpinen et al., 2013; McVicker et al., 2013). Here we show that there is variability on SNP linked to epigenetic genes within cattle. This combined with the fact that, at least some, epigenetic processes can be modulated by DNA variants, opens new opportunities for further research. Genome-wide association studies in this context are still to be seen.

Epigenetics is a very dynamic field of research and the list of genes and transcripts involved with epigenetic processes is growing. The ongoing gene annotation process means that the list of genes considered here should be considered only as a starting point. The analysis presented here are intended as a framework for mining epigenetic information from SNP panels and in any new analysis the list of target genes and transcripts should be revisited to include the most recent annotation as well as the most recent assembly of the investigated genome.

We conclude that there is variation on SNP connected to epigenetic processes between cattle breeds. This variation can be used to differentiate cattle breeds. Further investigation would confirm if SNP located near or in genes of the epigenetic machinery are useful to “tag” biological variation associated to epigenetic processes.

## Author contributions

Laercio R. Porto-Neto and Antonio Reverter designed the experiment. Laercio R. Porto-Neto, Marina R. S. Fortes, Sean M. McWilliam, Sigrid A. Lehnert, and Antonio Reverter contributed to data acquisition. Laercio R. Porto-Neto and Sean M. McWilliam contributed to data mining and bioinformatic analyses. Laercio R. Porto-Neto performed population diversity and statistical analysis. Laercio R. Porto-Neto, Marina R. S. Fortes, Sigrid A. Lehnert, and Antonio Reverter contributed to interpretation of results and drafted the manuscript. All authors approved the final version of the manuscript.

### Conflict of interest statement

The authors declare that the research was conducted in the absence of any commercial or financial relationships that could be construed as a potential conflict of interest.
