# Supplementary material for: Variation in genes involved in epigenetic processes offers insights into tropically adapted cattle diversity
Source: Front Genet. 2014 Apr 22;5:89. doi: 10.3389/fgene.2014.00089 (PMC4001012; doi:10.3389/fgene.2014.00089)
Supplement: Supplementary file 1 [file DataSheet1.DOCX]

**In Research Topic: Understanding livestock epigenetics towards a more sustainable animal production systems, 2014.**

***Supplementary Material:***

**Variation in genes involved in epigenetic processes offers insights into tropically adapted cattle diversity**

Laercio R. Porto-Neto^1^, Marina R.S. Fortes^2^, Sean M. McWilliam^1^, Sigrid A. Lehnert^1^, Antonio Reverter^1*^

^1^ CSIRO Food Futures Flagship and CSIRO Animal, Health and Food Sciences, Brisbane, Queensland, Australia.

^2^ The University of Queensland, QAAFI Centre for Animal Science, Brisbane, Queensland, Australia.

* **Correspondence:** Dr. Antonio Reverter, CSIRO Food Futures Flagship and CSIRO Animal, Health and Food Sciences, 306 Carmody Road, St Lucia, QLD 4067, Australia.

Toni.Reverter-Gomez@csiro.au

1. **Supplementary Table**

**Supplementary table1. List of epigenetic transcripts tagged by BovineHD SNP.**

Epigenetic category: a - DNA methyltransferases (DNMT), b - Methyl-binding domain proteins (MBD), c - Histone acetyltransferases (HAT), d - Histone deacetylases (HDAC), e - Histone methyltransferases, (HMT), f - Histone demethylase, g - Histones, h - Polycomb-group proteins, i - Chromatin-remodelling factors. CAT1 = SNP at transcribed regions (exon, splice region, 3’- and 5’-UTR); CAT2 = SNP at transcripts intron; CAT3 = SNP down- or up-stream of a transcript (up to 3Kb).

| **ENSBTAG** | **BTA** | **Epigenetic category** | **Total number of SNP** | **CAT1** | **CAT2** | **CAT3** |
| --- | --- | --- | --- | --- | --- | --- |
| ENSBTAG00000002736 | 7 | a | 15 | 2 | 9 | 4 |
| ENSBTAG00000021143 | 11 | a | 6 | 0 | 5 | 1 |
| ENSBTAG00000020546 | 13 | a | 20 | 0 | 19 | 1 |
| ENSBTAG00000038409 | 7 | b | 2 | 0 | 0 | 2 |
| ENSBTAG00000013236 | 19 | b | 2 | 0 | 2 | 0 |
| ENSBTAG00000003801 | 24 | b | 5 | 0 | 4 | 1 |
| ENSBTAG00000037457 | 24 | b | 12 | 0 | 10 | 2 |
| ENSBTAG00000007236 | 27 | b | 8 | 1 | 7 | 0 |
| ENSBTAG00000047855 | X | b | 24 | 1 | 20 | 3 |
| ENSBTAG00000002295 | 2 | c | 26 | 0 | 25 | 1 |
| ENSBTAG00000010118 | 2 | c | 7 | 0 | 6 | 1 |
| ENSBTAG00000016198 | 5 | c | 9 | 1 | 8 | 0 |
| ENSBTAG00000027764 | 10 | c | 1 | 0 | 0 | 1 |
| ENSBTAG00000004091 | 11 | c | 7 | 1 | 5 | 1 |
| ENSBTAG00000007489 | 13 | c | 17 | 1 | 15 | 1 |
| ENSBTAG00000015644 | 18 | c | 2 | 1 | 0 | 1 |
| ENSBTAG00000018699 | 19 | c | 21 | 1 | 18 | 2 |
| ENSBTAG00000016397 | 23 | c | 49 | 0 | 48 | 1 |
| ENSBTAG00000013917 | 25 | c | 10 | 2 | 5 | 3 |
| ENSBTAG00000026403 | 25 | c | 36 | 1 | 34 | 1 |
| ENSBTAG00000012698 | 2 | d | 9 | 1 | 7 | 1 |
| ENSBTAG00000017764 | 3 | d | 161 | 0 | 161 | 0 |
| ENSBTAG00000003808 | 4 | d | 123 | 0 | 121 | 2 |
| ENSBTAG00000011000 | 5 | d | 1 | 0 | 1 | 0 |
| ENSBTAG00000026819 | 5 | d | 8 | 0 | 7 | 1 |
| ENSBTAG00000011849 | 9 | d | 15 | 1 | 12 | 2 |
| ENSBTAG00000016254 | 19 | d | 9 | 0 | 8 | 1 |
| ENSBTAG00000014023 | 28 | d | 3 | 0 | 1 | 2 |
| ENSBTAG00000021253 | 1 | e | 20 | 1 | 17 | 2 |
| ENSBTAG00000001357 | 2 | e | 1 | 1 | 0 | 0 |
| ENSBTAG00000000098 | 3 | e | 11 | 0 | 11 | 0 |
| ENSBTAG00000009426 | 4 | e | 11 | 0 | 10 | 1 |
| ENSBTAG00000024199 | 4 | e | 49 | 3 | 44 | 2 |
| ENSBTAG00000009447 | 5 | e | 22 | 1 | 21 | 0 |
| ENSBTAG00000014429 | 5 | e | 10 | 4 | 5 | 1 |
| ENSBTAG00000007986 | 6 | e | 11 | 0 | 10 | 1 |
| ENSBTAG00000012111 | 6 | e | 64 | 0 | 64 | 0 |
| ENSBTAG00000020570 | 6 | e | 3 | 0 | 1 | 2 |
| ENSBTAG00000012048 | 7 | e | 6 | 0 | 5 | 1 |
| ENSBTAG00000020578 | 7 | e | 67 | 1 | 65 | 1 |
| ENSBTAG00000025426 | 7 | e | 24 | 1 | 23 | 0 |
| ENSBTAG00000000816 | 9 | e | 9 | 0 | 6 | 3 |
| ENSBTAG00000006095 | 9 | e | 4 | 0 | 2 | 2 |
| ENSBTAG00000010890 | 10 | e | 3 | 0 | 2 | 1 |
| ENSBTAG00000012148 | 11 | e | 21 | 1 | 18 | 2 |
| ENSBTAG00000023797 | 11 | e | 4 | 0 | 2 | 2 |
| ENSBTAG00000010210 | 13 | e | 7 | 0 | 6 | 1 |
| ENSBTAG00000000060 | 14 | e | 5 | 0 | 4 | 1 |
| ENSBTAG00000006443 | 15 | e | 18 | 0 | 17 | 1 |
| ENSBTAG00000018093 | 15 | e | 12 | 0 | 11 | 1 |
| ENSBTAG00000018965 | 16 | e | 14 | 2 | 12 | 0 |
| ENSBTAG00000020552 | 16 | e | 18 | 1 | 15 | 2 |
| ENSBTAG00000033180 | 16 | e | 222 | 0 | 220 | 2 |
| ENSBTAG00000003581 | 17 | e | 13 | 2 | 11 | 0 |
| ENSBTAG00000006646 | 18 | e | 3 | 1 | 2 | 0 |
| ENSBTAG00000014087 | 18 | e | 5 | 1 | 0 | 4 |
| ENSBTAG00000021918 | 19 | e | 6 | 3 | 2 | 1 |
| ENSBTAG00000003957 | 21 | e | 5 | 1 | 3 | 1 |
| ENSBTAG00000005676 | 23 | e | 6 | 1 | 4 | 1 |
| ENSBTAG00000019001 | 29 | e | 26 | 1 | 24 | 1 |
| ENSBTAG00000012052 | 2 | f | 14 | 0 | 12 | 2 |
| ENSBTAG00000040155 | 1 | g | 1 | 0 | 0 | 1 |
| ENSBTAG00000005904 | 2 | g | 4 | 0 | 4 | 0 |
| ENSBTAG00000032451 | 3 | g | 1 | 0 | 0 | 1 |
| ENSBTAG00000032453 | 3 | g | 2 | 0 | 0 | 2 |
| ENSBTAG00000032455 | 3 | g | 1 | 0 | 0 | 1 |
| ENSBTAG00000038604 | 3 | g | 2 | 0 | 0 | 2 |
| ENSBTAG00000039951 | 3 | g | 3 | 0 | 0 | 3 |
| ENSBTAG00000030504 | 5 | g | 1 | 0 | 0 | 1 |
| ENSBTAG00000004428 | 6 | g | 1 | 0 | 0 | 1 |
| ENSBTAG00000024561 | 16 | g | 4 | 0 | 2 | 2 |
| ENSBTAG00000019757 | 17 | g | 9 | 1 | 7 | 1 |
| ENSBTAG00000024909 | 19 | g | 1 | 0 | 0 | 1 |
| ENSBTAG00000011677 | 23 | g | 2 | 0 | 0 | 2 |
| ENSBTAG00000024177 | 23 | g | 1 | 0 | 0 | 1 |
| ENSBTAG00000024178 | 23 | g | 1 | 0 | 0 | 1 |
| ENSBTAG00000024179 | 23 | g | 1 | 0 | 0 | 1 |
| ENSBTAG00000024188 | 23 | g | 1 | 0 | 0 | 1 |
| ENSBTAG00000025391 | 23 | g | 1 | 0 | 0 | 1 |
| ENSBTAG00000031756 | 23 | g | 3 | 0 | 0 | 3 |
| ENSBTAG00000031762 | 23 | g | 2 | 0 | 0 | 2 |
| ENSBTAG00000031763 | 23 | g | 1 | 0 | 0 | 1 |
| ENSBTAG00000031768 | 23 | g | 1 | 0 | 0 | 1 |
| ENSBTAG00000031769 | 23 | g | 3 | 0 | 0 | 3 |
| ENSBTAG00000031770 | 23 | g | 2 | 0 | 0 | 2 |
| ENSBTAG00000031785 | 23 | g | 3 | 0 | 3 | 0 |
| ENSBTAG00000031886 | 23 | g | 4 | 1 | 2 | 1 |
| ENSBTAG00000031889 | 23 | g | 1 | 0 | 0 | 1 |
| ENSBTAG00000034582 | 23 | g | 2 | 0 | 0 | 2 |
| ENSBTAG00000039812 | 23 | g | 3 | 1 | 0 | 2 |
| ENSBTAG00000046621 | 23 | g | 2 | 0 | 0 | 2 |
| ENSBTAG00000014017 | 6 | h | 9 | 0 | 7 | 2 |
| ENSBTAG00000015584 | 13 | h | 3 | 0 | 0 | 3 |
| ENSBTAG00000004964 | 26 | h | 8 | 0 | 7 | 1 |
| ENSBTAG00000000746 | 1 | i | 32 | 0 | 31 | 1 |
| ENSBTAG00000002460 | 1 | i | 8 | 0 | 8 | 0 |
| ENSBTAG00000016619 | 1 | i | 6 | 0 | 2 | 4 |
| ENSBTAG00000018682 | 1 | i | 14 | 2 | 11 | 1 |
| ENSBTAG00000019704 | 1 | i | 23 | 0 | 21 | 2 |
| ENSBTAG00000001024 | 2 | i | 24 | 1 | 23 | 0 |
| ENSBTAG00000003843 | 2 | i | 31 | 2 | 25 | 4 |
| ENSBTAG00000016334 | 2 | i | 57 | 2 | 55 | 0 |
| ENSBTAG00000038104 | 2 | i | 2 | 0 | 0 | 2 |
| ENSBTAG00000001393 | 3 | i | 13 | 0 | 11 | 2 |
| ENSBTAG00000001629 | 3 | i | 43 | 2 | 38 | 3 |
| ENSBTAG00000002078 | 3 | i | 10 | 2 | 8 | 0 |
| ENSBTAG00000003724 | 3 | i | 4 | 0 | 3 | 1 |
| ENSBTAG00000003954 | 3 | i | 55 | 3 | 50 | 2 |
| ENSBTAG00000015346 | 3 | i | 4 | 0 | 3 | 1 |
| ENSBTAG00000020831 | 3 | i | 40 | 0 | 38 | 2 |
| ENSBTAG00000030710 | 3 | i | 14 | 0 | 14 | 0 |
| ENSBTAG00000002256 | 5 | i | 15 | 1 | 12 | 2 |
| ENSBTAG00000002492 | 5 | i | 4 | 0 | 2 | 2 |
| ENSBTAG00000006246 | 5 | i | 8 | 0 | 6 | 2 |
| ENSBTAG00000014697 | 5 | i | 4 | 0 | 3 | 1 |
| ENSBTAG00000014734 | 5 | i | 14 | 4 | 10 | 0 |
| ENSBTAG00000017840 | 5 | i | 7 | 2 | 3 | 2 |
| ENSBTAG00000018205 | 5 | i | 7 | 1 | 5 | 1 |
| ENSBTAG00000037935 | 5 | i | 4 | 0 | 4 | 0 |
| ENSBTAG00000004191 | 7 | i | 4 | 1 | 1 | 2 |
| ENSBTAG00000006083 | 7 | i | 3 | 0 | 2 | 1 |
| ENSBTAG00000008789 | 7 | i | 2 | 0 | 0 | 2 |
| ENSBTAG00000010063 | 7 | i | 11 | 0 | 11 | 0 |
| ENSBTAG00000015487 | 7 | i | 37 | 0 | 36 | 1 |
| ENSBTAG00000019220 | 7 | i | 32 | 1 | 31 | 0 |
| ENSBTAG00000020860 | 7 | i | 24 | 1 | 21 | 2 |
| ENSBTAG00000007494 | 8 | i | 71 | 1 | 68 | 2 |
| ENSBTAG00000015101 | 8 | i | 3 | 0 | 1 | 2 |
| ENSBTAG00000002728 | 9 | i | 110 | 2 | 106 | 2 |
| ENSBTAG00000004420 | 10 | i | 4 | 0 | 2 | 2 |
| ENSBTAG00000010380 | 10 | i | 27 | 1 | 25 | 1 |
| ENSBTAG00000010766 | 10 | i | 1 | 0 | 1 | 0 |
| ENSBTAG00000015148 | 10 | i | 2 | 1 | 1 | 0 |
| ENSBTAG00000020422 | 10 | i | 8 | 0 | 8 | 0 |
| ENSBTAG00000010582 | 11 | i | 4 | 0 | 3 | 1 |
| ENSBTAG00000013580 | 11 | i | 20 | 1 | 19 | 0 |
| ENSBTAG00000013589 | 11 | i | 11 | 1 | 9 | 1 |
| ENSBTAG00000015014 | 11 | i | 3 | 0 | 3 | 0 |
| ENSBTAG00000021120 | 11 | i | 22 | 1 | 20 | 1 |
| ENSBTAG00000006640 | 12 | i | 32 | 2 | 28 | 2 |
| ENSBTAG00000005733 | 13 | i | 67 | 3 | 61 | 3 |
| ENSBTAG00000004954 | 14 | i | 101 | 1 | 98 | 2 |
| ENSBTAG00000020226 | 14 | i | 2 | 0 | 1 | 1 |
| ENSBTAG00000016347 | 16 | i | 1 | 0 | 0 | 1 |
| ENSBTAG00000018272 | 16 | i | 49 | 0 | 49 | 0 |
| ENSBTAG00000040477 | 16 | i | 16 | 0 | 14 | 2 |
| ENSBTAG00000003399 | 17 | i | 6 | 0 | 5 | 1 |
| ENSBTAG00000009988 | 17 | i | 5 | 0 | 4 | 1 |
| ENSBTAG00000021795 | 17 | i | 7 | 1 | 6 | 0 |
| ENSBTAG00000000418 | 18 | i | 34 | 1 | 33 | 0 |
| ENSBTAG00000002763 | 18 | i | 4 | 1 | 3 | 0 |
| ENSBTAG00000010467 | 18 | i | 18 | 3 | 14 | 1 |
| ENSBTAG00000021575 | 18 | i | 7 | 0 | 7 | 0 |
| ENSBTAG00000003386 | 19 | i | 1 | 0 | 1 | 0 |
| ENSBTAG00000009036 | 19 | i | 7 | 1 | 4 | 2 |
| ENSBTAG00000011539 | 19 | i | 2 | 1 | 0 | 1 |
| ENSBTAG00000014377 | 19 | i | 5 | 1 | 4 | 0 |
| ENSBTAG00000014598 | 19 | i | 32 | 2 | 30 | 0 |
| ENSBTAG00000015308 | 19 | i | 8 | 1 | 5 | 2 |
| ENSBTAG00000006971 | 20 | i | 10 | 1 | 8 | 1 |
| ENSBTAG00000014246 | 20 | i | 4 | 1 | 3 | 0 |
| ENSBTAG00000044175 | 20 | i | 6 | 0 | 6 | 0 |
| ENSBTAG00000010988 | 21 | i | 6 | 0 | 3 | 3 |
| ENSBTAG00000013888 | 21 | i | 2 | 0 | 1 | 1 |
| ENSBTAG00000020164 | 21 | i | 9 | 1 | 7 | 1 |
| ENSBTAG00000020819 | 21 | i | 8 | 0 | 7 | 1 |
| ENSBTAG00000014786 | 22 | i | 21 | 1 | 20 | 0 |
| ENSBTAG00000020998 | 22 | i | 14 | 0 | 12 | 2 |
| ENSBTAG00000031567 | 22 | i | 45 | 0 | 41 | 4 |
| ENSBTAG00000020710 | 23 | i | 6 | 0 | 5 | 1 |
| ENSBTAG00000002345 | 25 | i | 7 | 2 | 5 | 0 |
| ENSBTAG00000005197 | 25 | i | 14 | 2 | 12 | 0 |
| ENSBTAG00000005979 | 26 | i | 13 | 2 | 10 | 1 |
| ENSBTAG00000017804 | 26 | i | 2 | 0 | 0 | 2 |
| ENSBTAG00000000978 | 27 | i | 9 | 0 | 5 | 4 |
| ENSBTAG00000004015 | 27 | i | 19 | 0 | 19 | 0 |
| ENSBTAG00000018864 | 27 | i | 7 | 0 | 7 | 0 |
| ENSBTAG00000001573 | 28 | i | 75 | 0 | 74 | 1 |
| ENSBTAG00000001185 | 29 | i | 11 | 0 | 9 | 2 |
| ENSBTAG00000007847 | 29 | i | 9 | 0 | 8 | 1 |
| ENSBTAG00000009484 | 29 | i | 4 | 0 | 3 | 1 |
| ENSBTAG00000011865 | 29 | i | 3 | 0 | 3 | 0 |
| ENSBTAG00000017689 | 29 | i | 2 | 0 | 2 | 0 |
| ENSBTAG00000020268 | 29 | i | 17 | 1 | 15 | 1 |
| ENSBTAG00000001311 | X | i | 8 | 0 | 7 | 1 |
| ENSBTAG00000002292 | X | i | 19 | 1 | 15 | 3 |
| ENSBTAG00000002820 | X | i | 21 | 1 | 17 | 3 |
| ENSBTAG00000004706 | X | i | 9 | 1 | 5 | 3 |
| ENSBTAG00000005150 | X | i | 3 | 0 | 2 | 1 |
| ENSBTAG00000009278 | X | i | 8 | 1 | 5 | 2 |
| ENSBTAG00000009420 | X | i | 28 | 1 | 26 | 1 |
| ENSBTAG00000013279 | X | i | 5 | 0 | 4 | 1 |
| ENSBTAG00000021254 | X | i | 15 | 3 | 12 | 0 |
